# Supplementary material for: Considering distributive justice as a planning principle helps navigate a diversity of future energy infrastructure designs
Source: Nat Commun. 2025 Nov 25;16:10509. doi: 10.1038/s41467-025-65526-0 (PMC12647790; doi:10.1038/s41467-025-65526-0)
Supplement: Supplementary file 1 — Supplementary Information [file 41467_2025_65526_MOESM1_ESM.pdf]

Supplementary information for "Considering distributive justice as a planning principle helps navigate a diversity of future energy infrastructure designs"

Katherine Emma Lonergan[1,2] and Giovanni Sansavini[1,2 †]

[1]Reliability and Risk Engineering, ETH Zurich, 8092 Zurich, Switzerland
[2]Institute of Energy and Process Engineering, ETH Zurich, 8092 Zurich, Switzerland

†Corresponding author: Giovanni Sansavini (sansavig@ethz.ch)

Table of Tables

Supplementary Table 1. Justice indicators and corresponding policy examples 4

Supplementary Table 2. Range reduction potential from considering top 1%, 5%, and 10% of top-performing candidate designs. 6

Supplementary Table 3. Summary preference ranges for distributive justice theory and impacts 6

Supplementary Table 4. Preferences for justice theories expressed in Eurobarometer surveys. 6

Supplementary Table 5. Preferences for impact categories as expressed in Eurobarometer surveys...... 7

Supplementary Table 6. Sensitivity analysis results for range reduction. 7

Table of Figures

Supplementary Figure 1.Absolute annual **a** investment, **b** jobs, and **c** land use according to the candidate designs providing the most equal investment, job, and land use distribution, respectively. ... 8

Supplementary Figure 2.Absolute annual **a** investment, **b** jobs, and **c** land use according to the candidate designs providing the most equitable investment, job, and land use distribution, respectively. 8

Supplementary Figure 3. Distribution of best-performing candidate designs according to the equal distribution of investment, job, and land use when identifying the best-performing candidate design according to minimising the total absolute percent difference from the ideal distribution (**a, c, e**) and when minimising the maximum percent difference (**b, d, f**) for individual countries.. 9

Supplementary Figure 4. Heatmap of correlations between justice indicators. 10

Supplementary Figure 5. Overall trade-offs between justice approaches and investment costs, job creation, and land use for candidate designs. 11

Supplementary Figure 6. Trade-offs between job creation and land use with varying assumptions on PV land-use requirements.. 12

Supplementary Figure 7. Cumulative distribution function (CDF) of the share candidate designs ranked within the top percentile of different distributive justice indicators. 12

Supplementary Figure 8. Key infrastructure for the top 1% of candidate designs (4 designs) according to indicator 8, maximum job creation (utility). 13

Supplementary Figure 9. Key infrastructure for the top 1% of candidate designs (4 designs) according to indicator 1, equality of investment.. 14

Supplementary Figure 10. Key infrastructure for the top 1% of candidate designs (4 designs) according to indicator 2, equality of job creation. 15

Supplementary Figure 11. Key infrastructure for the top 1% of candidate designs (4 designs) according to indicator 3, equality of land use. 16

Supplementary Figure 12. Key infrastructure for the top 1% of candidate designs (4 designs) according to indicator 4, equitable investment. ... 17

Supplementary Figure 13. Key infrastructure for the top 1% of candidate designs (4 designs) according to indicator 5, equitable job creation. ... 18

Supplementary Figure 14. Key infrastructure for the top 1% of candidate designs (4 designs) according to indicator 6, equitable land use. ... 19

Supplementary Figure 15. Key infrastructure for the top 1% of candidate designs (4 designs) according to indicator 7, minimum investment (utility). ... 20

Supplementary Figure 16. Key infrastructure for the top 1% of candidate designs (4 designs) according to indicator 9, minimum land use (utility). ... 21

Supplementary Figure 17. Ranked importance of onshore wind capacity versus other key technologies across the justice-ranked candidate designs. high shares of onshore wind do not necessarily signal just energy systems.. ... 22

Supplementary Figure 18. Onshore wind capacity distribution of top 10% most just candidate designs according to the nine indicators for distributive justice and the BP approach. ... 23

Supplementary Figure 19. Sensitivity analysis on design space reduction by technology. ... 24

Supplementary Figure 20. Cost distribution of 10% best-scoring BP candidate designs versus all other candidate designs. ... 25

Supplementary Figure 21. Per capita impacts of all 353 unique candidate designs ... 25

Supplementary Table 1. Justice indicators and corresponding policy examples. Strategic European technologies are heat pumps, direct air capture, batteries, solar photovoltaic, on- and offshore wind. See Methods – Justice Indicators.

Indicator	Goal	Example European policies
Equality – Investment	Distribute investment equally on a per capita basis (national)	• Directive (EU) 2019/944 (on common rules for the electricity market)<br>• COM(2015) 80 (Energy Union Package): 1. "Building up investment in high-tech, globally competing companies through stable policies will bring jobs and growth to Europe. New business sectors, new business models and new job profiles will emerge. Such transformational change profoundly affects the roles of all actors in the energy system, including the consumers. Europe needs to make the right choices now. If it continues on the present path, the unavoidable challenge of shifting to a low-carbon economy will be made harder by the economic, social and environmental costs of having fragmented national energy markets."
Equality – Jobs	Provide equal opportunities to jobs in key technologies on a per capita basis (national)	• European Pillar of Social Rights Action Plan (2021) 3. Equal opportunities<br>• European Pillar of Social Rights Action Plan (2021) 5. Secure and adaptable employment<br>• COM(2019) 640 (The European Green Deal) "The transition is an opportunity to expand sustainable and job-intensive economic activity …However, the transformation is taking place at a too slow pace with progress neither widespread nor uniform. The European Green Deal will support and accelerate the EU's industry transition to a sustainable model of inclusive growth."<br>• European Skills Agenda (2020) 7. Increasing STEM graduates and fostering entrepreneurial and transversal skills.
Equality – Land use	Distribute remaining land use requirements equally based on remaining available land (national)	• COM(2020) 380 (EU Biodiversity Strategy for 2030) 2.1 "The targets relate to the EU as a whole and could be broken down according to the EU bio-geographical regions and sea basins or at a more local level. Every Member State will have to do its fair share of the effort based on objective ecological criteria…"
Equity – Investment	Distribute investment to ensure all achieve the same benefits and equal protections from the low-carbon transition	• Directive (EU) 2023/1791 (Energy Efficiency) 24.1 "Member States shall take appropriate measures to empower and protect people affected by energy poverty, vulnerable customers, people in low-income households and, where applicable, people living in social housing."<br>• Directive (EU) 2023/1791 (Energy Efficiency) 24.3(c) "To support people affected by energy poverty, vulnerable customers, people in low-income households and, where applicable, people living in social housing, Member States shall, where applicable… carry out early, forward-looking investments in energy efficiency improvement measures before distributional impacts from other policies and measures show their effect"<br>• Regulation (EU) 2021/1058 (European Regional Development Fund and the Cohesion Fund) 2 "1. The ERDF and the Cohesion Fund shall contribute to the overall objective of strengthening the economic, social and territorial cohesion of the Union. 2. The ERDF shall contribute to reducing disparities between the levels of development of the various regions within the Union, and to reducing the backwardness of the least favoured regions through participation in the structural adjustment of regions whose development is lagging behind and in the conversion of declining industrial regions, including by promoting sustainable development and addressing environmental challenges. 3. The Cohesion Fund shall contribute to projects in the field of environment and trans-European networks in the area of transport infrastructure (TEN-T)."
Equity – Jobs	Distribute jobs in key technologies proportionally to the current distribution of jobs in the fossil fuel sector	• Regulation (EU) 2021/1056 (Just Transition Fund) "Member States shall prepare, together with the relevant local and regional authorities of the territories concerned, one or more territorial just transition…Those territories shall be those most negatively affected, based on the economic and social impacts resulting from the transition, in particular with regard to the expected adaptation of workers or job losses in fossil fuel production and use and the transformation needs of the production processes of industrial facilities with the highest greenhouse gas intensity."<br>• European Skills Agenda (2020) 5.1 "Investment in the quality, equity and labour market relevance of education and training systems to ensure that

Indicator	Goal	Example European policies
		people are equipped with the key competences needed in the labour market and in society.”<br>• COM(2023) 62 (Green Deal Industrial Plan) 2.3 “The green transition must be people-centred and inclusive to ensure equitable and just outcomes, generating quality jobs and leaving no-one behind… This is why the third pillar of the Green Deal Industrial Plan must focus on skills - green and digital, at all levels and for all people, with inclusiveness of women and youth at the heart of the Plan.”
Equity – Land use	Distribute land use requirements to ensure all nations achieve the same benefits and equal protections from the low-carbon transition	• Decision (EU) 2022/591 (General Union Environment Action Programme to 2030) 1.2 “The 8th EAP aims to accelerate the green transition to a climate-neutral, sustainable, non-toxic, resource-efficient, renewable energy-based, resilient and competitive circular economy in a just, equitable and inclusive way, and to protect, restore and improve the state of the environment by, inter alia, halting and reversing biodiversity loss. It supports and strengthens an integrated policy and implementation approach, building upon the European Green Deal.”<br>• Decision (EU) 2022/591 (General Union Environment Action Programme to 2030) 3 “The attainment of the priority objectives set out in Article 2 shall require the following from the Commission, Member States, regional and local authorities and stakeholders, as appropriate… prioritising enforcement of Union environmental law where implementation is lacking, including through infringement proceedings, as well as by ensuring that sufficient financial and human resources are allocated for that purpose and that information on those proceedings is complete and easily accessible, while respecting Union law…”
Utility – Investment	Minimise the total investment and operation cost	• COM(2015) 80 (Energy Union Package) 1. “The goal of a resilient Energy Union with an ambitious climate policy at its core is to give EU consumers - households and businesses - secure, sustainable, competitive and affordable energy.”<br>• Directive (EU) 2019/944 (on common rules for the electricity market) 1. “Using the advantages of an integrated market, this Directive aims to ensure affordable, transparent energy prices and costs for consumers, a high degree of security of supply and a smooth transition towards a sustainable low-carbon energy system.”
Utility – Jobs	Maximise the number of jobs linked to strategic European technologies	• European Skills Agenda (2020) “The green transition requires investments in skills of people to increase the number of professionals who build and master green technologies, including digital, develop green products, services and business models, create innovative nature-based solutions and help minimise the environmental footprint of activities37. In addition, Europe will only become a climate neutral continent, a resource efficient society and a circular economy with an informed population and workforce that understands how to think and act green.”
Utility – Land use	Minimise the direct total area required to host energy technologies	• COM(2020) 380 (EU Biodiversity Strategy for 2030) 10. “This should be done by improving and widening our network of protected areas and by developing an ambitious EU Nature Restoration Plan.”<br>• Directive (EU) 2023/2413 (promotion of energy from renewable sources) Preamble 7. “The multiple use of space for renewable energy production and other land, inland water and sea uses, such as food production or nature protection or restoration, alleviates the constraints on land, inland water and sea use. In that context, spatial planning is an essential tool with which to identify and steer synergies for land, inland water and sea use at an early stage. Member States should explore, enable and favour multiple uses of the areas identified as a result of the spatial planning measures adopted. To that end, Member States should facilitate changes in land and sea use where required, provided that the different uses and activities are compatible with one another and can co-exist.”

Supplementary Table 2. Range reduction potential from considering top 1%, 5%, and 10% of top-performing candidate designs. Results summarise the mean range reduction potential six key technologies of PV, onshore wind, offshore wind, batteries, heat pumps, and electrolysers. Mean values are provided in parentheses.

Indicator	1%	5%	10%
Equality – Investment	96% (94%)	65% (65%)	65% (64%)
Equality – Jobs	94% (88%)	63% (65%)	53% (45%)
Equality – Land	75% (68%)	68% (61%)	68% (58%)
Equity – Investment	93% (82%)	84% (72%)	26% (34%)
Equity – Jobs	91% (84%)	41% (41%)	32% (34%)
Equity – Land	57% (61%)	44% (53%)	36% (42%)
Utility – Investment	97% (91%)	91% (83%)	59% (55%)
Utility – Jobs	39% (40%)	39% (40%)	28% (26%)
Utility – Land	74% (62%)	73% (61%)	72% (61%)
Average	79% (74%)	61% (60%)	49% (47%)

Supplementary Table 3. Summary preference ranges for distributive justice theory and impacts (based on Supplementary Table 4 and Supplementary Table 5). Reference values are used for finding the balanced priorities (BP) solution; minimum and maximum values used within the sensitivity analysis. Reference values correspond to mean for all categories except for investment cost, where median is used to reflect the numerous relevant questions.

Distributive justice theory	Minimum	Maximum	Reference
Equality	9	63	36
Equity	89	92	90.5
Utilitarian	87	90	88.5
Impact	Minimum	Maximum	Reference
Investment cost	37	93	90
Jobs	55	88	71.5
Land	45	49	47

Supplementary Table 4. Preferences for justice theories expressed in Eurobarometer surveys.[1,2]

Justice theory	Reference survey	Reference question	Percent agreement
Equality	SP527	QA16.3 Allocating a quota of energy to each citizen to ensure everyone makes their fair share of effort to tackle climate change	62
Equality	SP527	QA.7 Who in our country should particularly make more efforts to reduce their energy consumption? → No particular group	9
Equity	SP527	QA1.2 The green transition should not leave anyone behind	88
Equity	SP527	QA16.4 Subsidising people to help make their homes more energy efficient, especially poorer people and the most vulnerable households (insulation, clean heating and cooling, energy production units, etc.)	89
Equity	SP527	QA7 Who in our country should particularly make more efforts to reduce their energy consumption? → Wealth-based	87
Equity	SP492	QB5.2 To what extent do you agree or disagree with the following statements? It should be the EU's responsibility to… address energy poverty and ensure a fair transition so that no citizen or region is left behind	90
Utility	SP492	QB2.1 The EU must secure access to energy to all EU citizens	92
Utility	SP492	QB2.3 The EU must ensure access to affordable energy, e.g., ensure competitive market prices, in particular to reduce the number of people unable to pay their energy bills	89
Utility	SP492	QB2.4 Cooperation between European Member States should be further strengthened to give all Europeans access to secure, financially affordable and clean energy	91
Utility	SP492	QB9 Top priorities for the next decade (maximum of three answers) → Ensure that costs are as low as possible	37

Supplementary Table 5. Preferences for impact categories as expressed in Eurobarometer surveys.[1–3] Questions regarding energy costs are used to proxy public sentiment about investment costs as no questions directly pertaining to investments could be identified.

Impact category	Reference survey	Reference question	Percent agreement
Investment costs	SP527	QA18 Would you be willing to pay higher energy prices if that helps to speed up the green transition? → Not able or not willing	64
Investment costs	SP527	QA17.1 The level of energy prices for people in (COUNTRY) in general is very serious or fairly serious?	93
Investment costs	SP492	QB9 In your opinion, which of the following energy issues should the European Union tackle as a priority over the next 10 years? (MAX. 3 ANSWERS) → Ensure that costs are as low as possible	37
Investment costs	SP492	QB4.2 To what extent do you agree or disagree with the following statements? It should be the EU's responsibility to… encourage more investment in renewable energy	90
Investment costs	SP492	QB4.3 To what extent do you agree or disagree with the following statements? It should be the EU's responsibility to … encourage more investment in energy research and innovation	92
Jobs	SP527	QA10.2 Being in a job that contributes to advancing the green transition is important to you personally	55
Jobs	SP527	QA1.2 The green transition should not leave anyone behind	87
Land use	SP550	QB13.A Which of the following actions should the EU prioritise to protect nature? First? And then? → Ensure that nature is protected when planning new developments or infrastructure	45
Land use	SP550	QB2T In your opinion, which of the following actions would be the most effective way of tackling environmental problems? First? Second? Third? Fourth? → Restoring nature	49

Supplementary Table 6. Sensitivity analysis results for range reduction. The balanced priorities (BP) weightings represent the public preferences expressed in the Barometer surveys. "Equally weighted" considers no relative preference between impacts or distributive justice theories, and all indicators are equally weighted. "Within surveyed ranges" considers 1,000 variations on public preferences, with preferences for distributive justice scheme and impacts varied between ranges identified within the Barometer surveys. "Random weights" also considers 1,000 variations on public preferences, with preferences for distributive justice scheme and impact randomly sampled from a range of 0-1 (also see Methods – Sensitivity analysis).

Weighting scheme	Minimum range reduction	Maximum range reduction	Average range reduction
BP	-	-	89%
Equally weighted	-	-	89%
Within surveyed ranges	86%	92%	91%
Random weights	54%	95%	85%

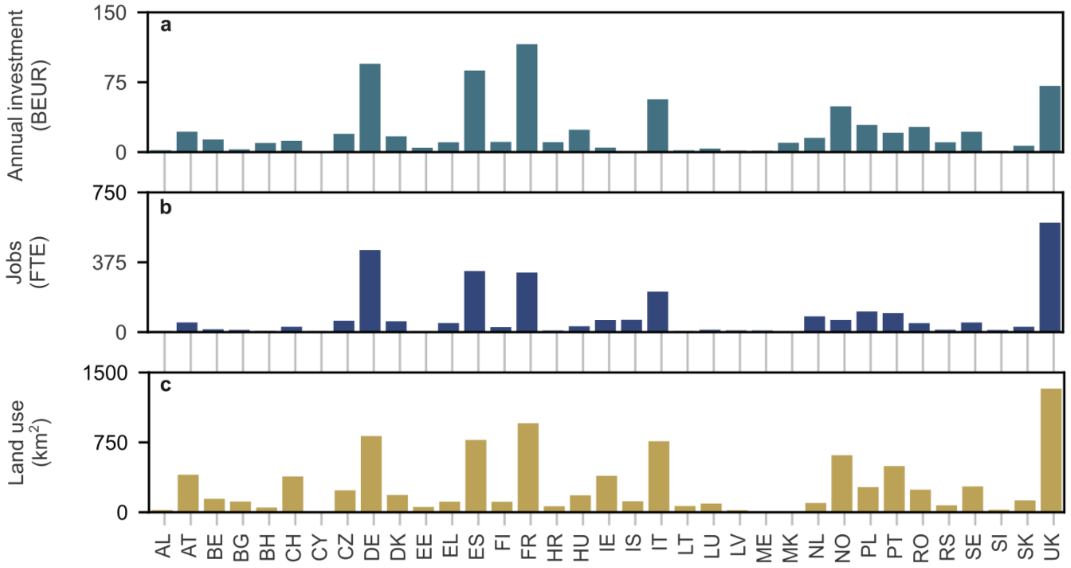

Supplementary Figure 1. Absolute investment, job, and land use according to the candidate designs providing the most equal impact distribution. Impact distribution for the most equal (a) investment, (b) job, and (c) land use. Although the relative job creation and land use in Iceland and Lithuania are high compared to other countries (Figure 2), the total job creation and land use are small in absolute terms.

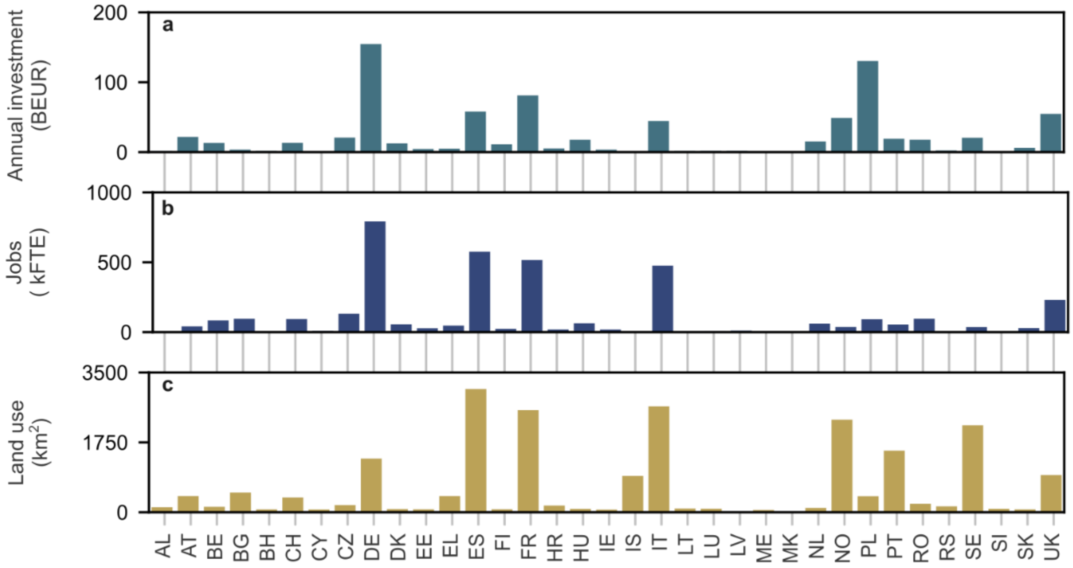

Supplementary Figure 2. Absolute investment, job, and land use according to the candidate designs providing the most equitable impact distribution. Impact distribution for the most equitable (a) investment, (b) job, and (c) land use. The row-to-row variation in distribution reflects the different equity basis for each indicator (the Cohesion Fund, current fossil fuel workers, and land protection targets; see Methods – Equity indicators).

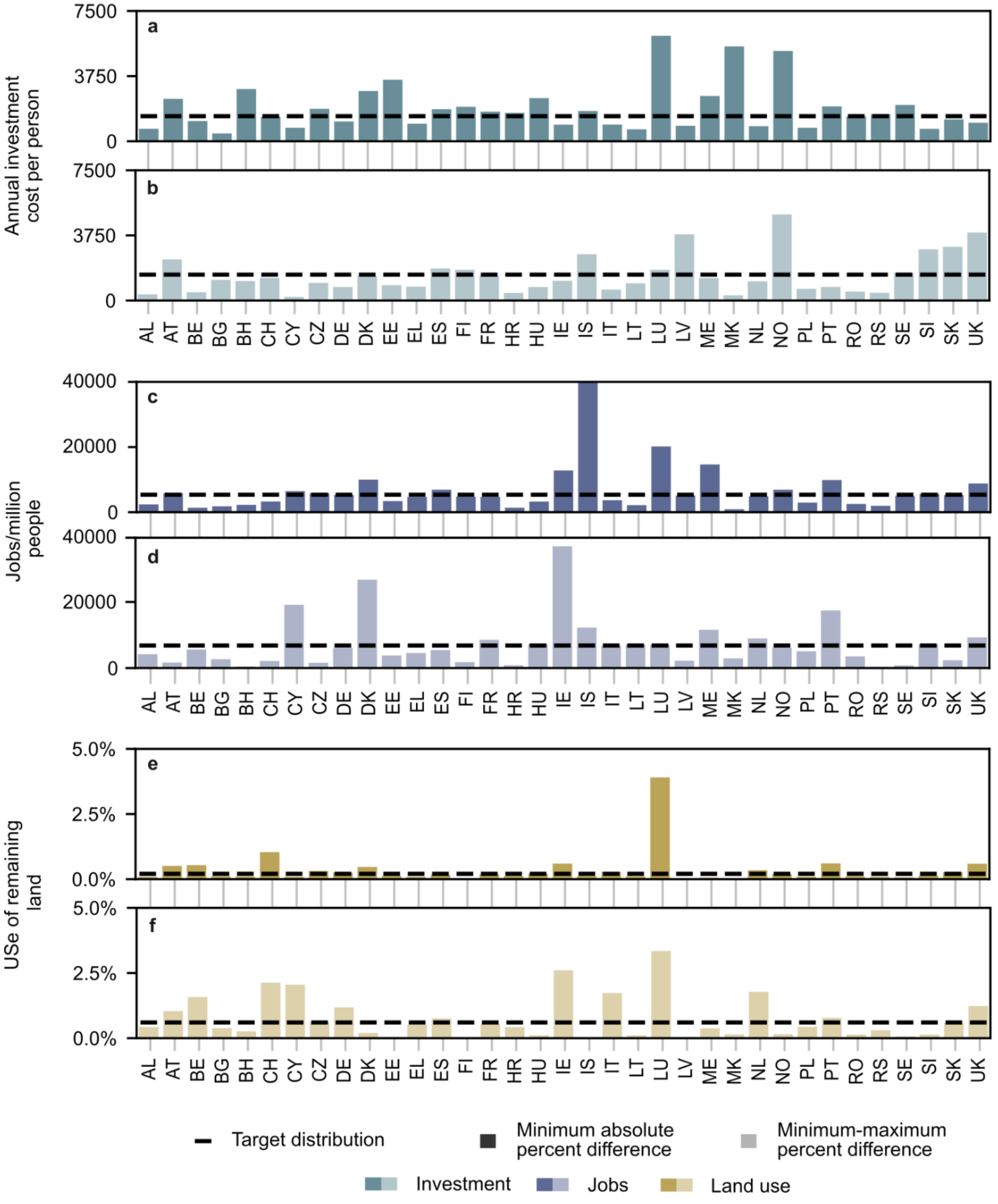

Supplementary Figure 3. Distribution of best-performing candidate designs according to the equal distribution of investment, job, and land use when identifying the best-performing candidate design according to minimising the total absolute percent difference from the ideal distribution (a, c, e) and when minimising the maximum percent difference (b, d, f) for individual countries. The latter approach helps equalize the burden/benefit sharing across countries and can therefore present a more politically viable option than the former approach.

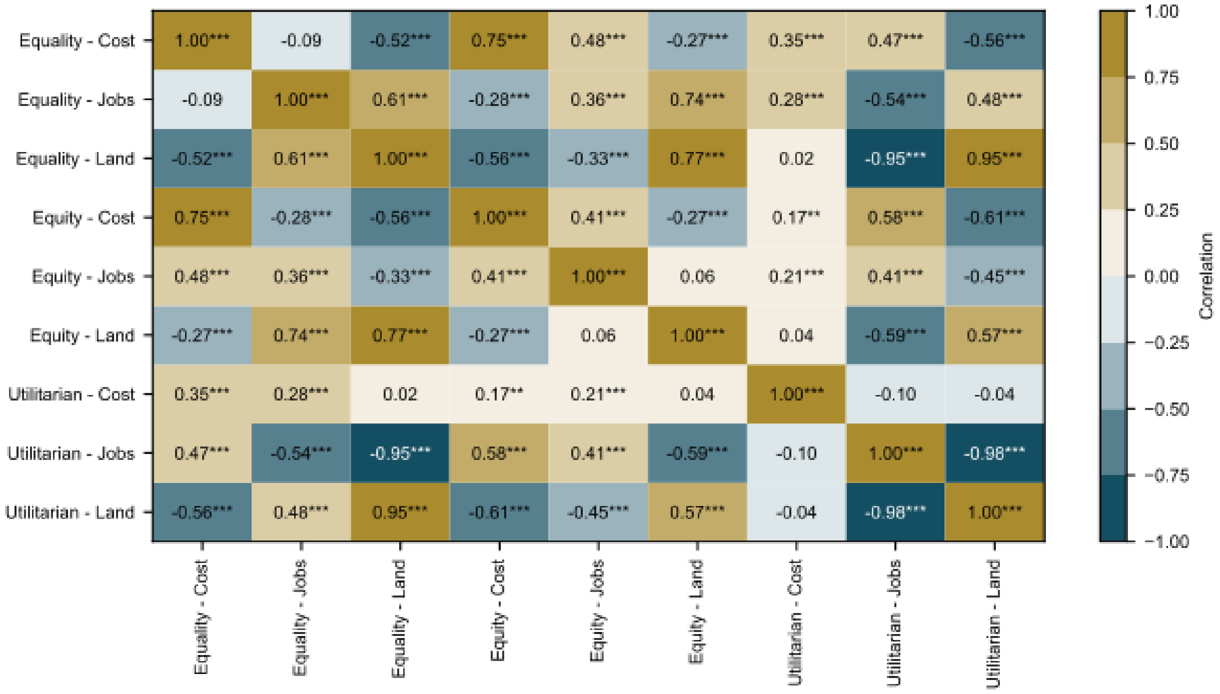

Supplementary Figure 4. Heatmap of linear correlations between justice indicators. Significance indicated for 95% (*), 99% (**), and 99.9% (***) levels (n=353). The strongest correlations are 0.98 between indicators for utility-based job and land distribution. This correlation is explained by the high employment factors and low land burden associated with rooftop solar PV. The two indicators also exhibit high correlation with equal land distribution. Other indicator pairings with high correlation include equal and equitable land use and equitable and equal cost. Least cost solution (Utilitarian – cost) show the lowest absolute correlations with other indicators. Correlations were calculated using scipy.stats module in Python[4].

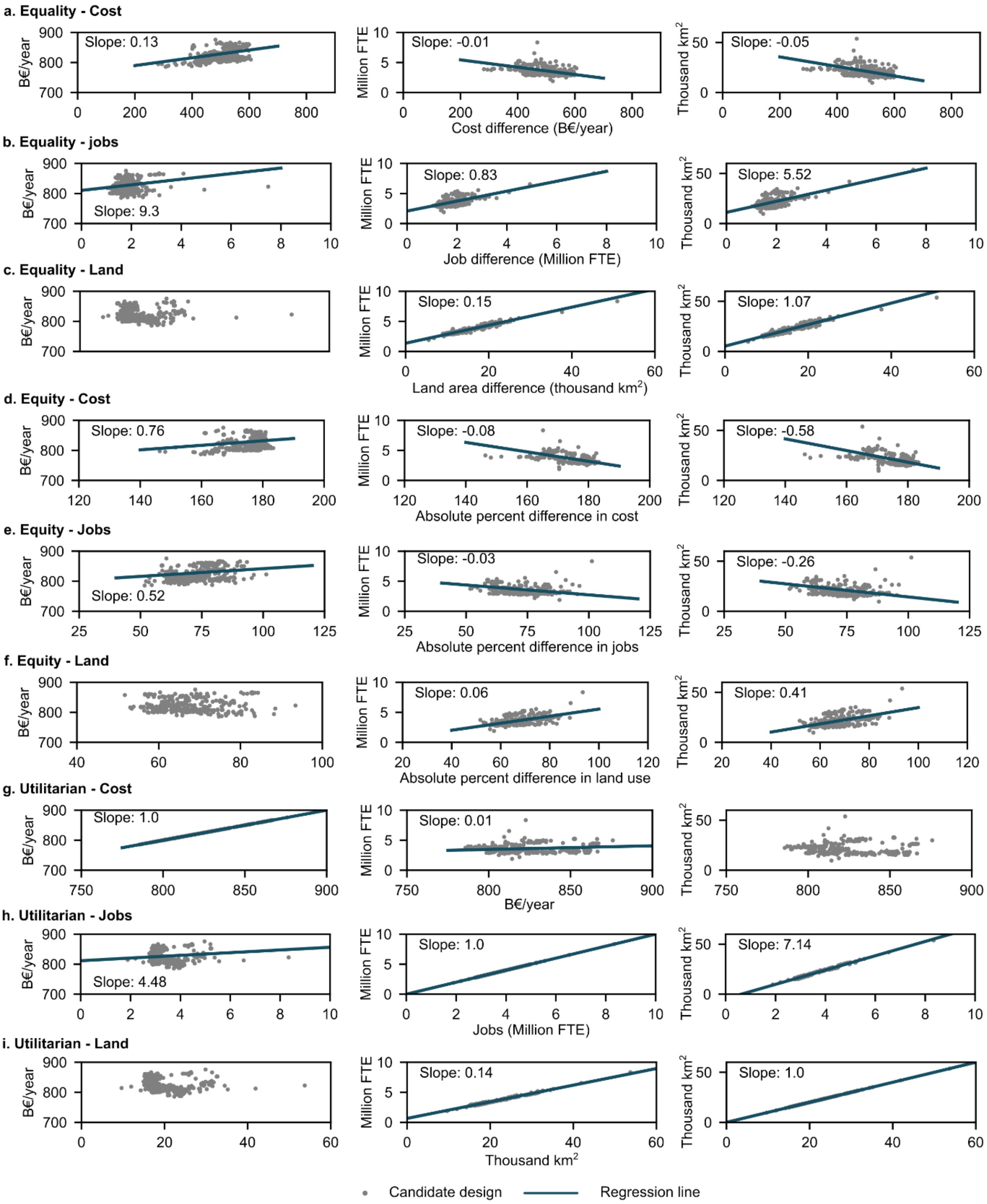

Supplementary Figure 5. Overall trade-offs between justice approaches and investment costs, job creation, and land use for candidate designs (n=353). The most just candidate designs according to each indicator (a)-(i) are the left-most candidate designs, except for h where the most just solution results in more job creation. Absolute job creation and land use are related so strongly given the high job creation potential and land use requirements associated with open field PV. Relying only on rooftop PV or increasing the production (Supplementary Fig. 6) intensity facilitates greater job creation with less land use. Regression lines shown when p-values < 0.05; full regression results available in Supplementary Data[5].

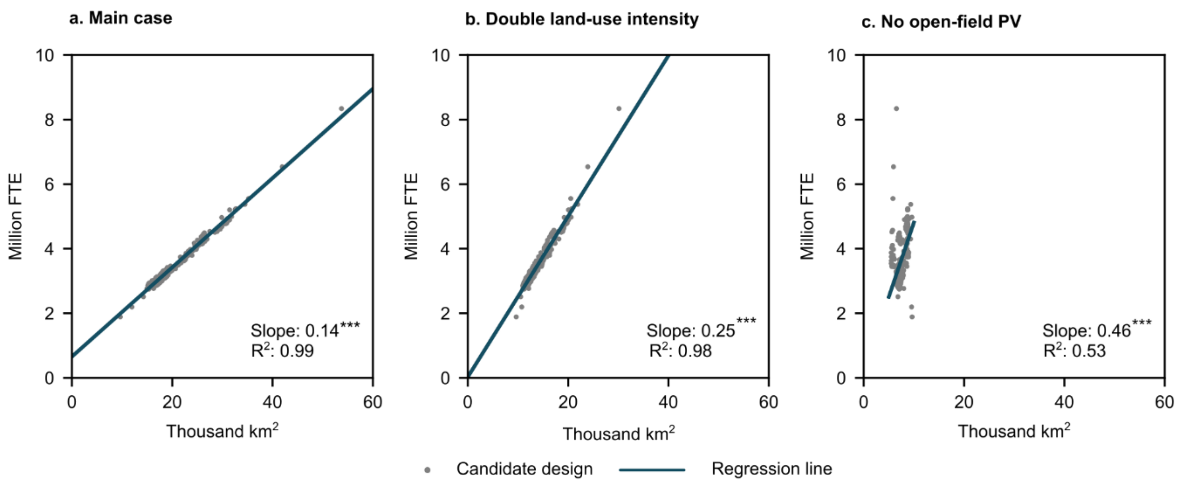

Supplementary Figure 6. Trade-offs between job creation and land use with varying assumptions on PV land-use requirements (n=353). Doubling the land use requirements (b) reduces the total land use requirements by roughly 20 thousand km^2 and weakens the relationship between land use and job creation. Preventing open field PV (c), e.g., by shifting entirely to rooftop and façade installations, suggests that the 460 jobs can be created per km^2 of land use, nearly a factor of 3 increase over the main case (a). All land use requirements sourced the ecoinvent database[6]. ***: p-value<0.001. All results calculated using the scipy.stats module in Python[4].

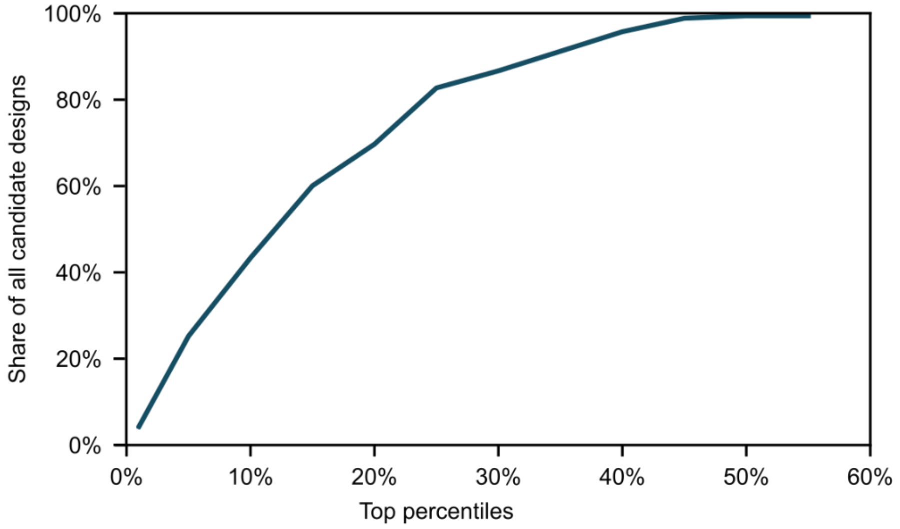

Supplementary Figure 7. Cumulative distribution function (CDF) of the share candidate designs ranked within the top percentile of different distributive justice indicators (n=353). There are always more candidate designs in the top percentiles than the theoretical minimum, highlighting that the best candidate designs vary according to justice indicator. 99% of all candidate designs are in the top-half of ranked solutions according to at least one distributive justice indicator.

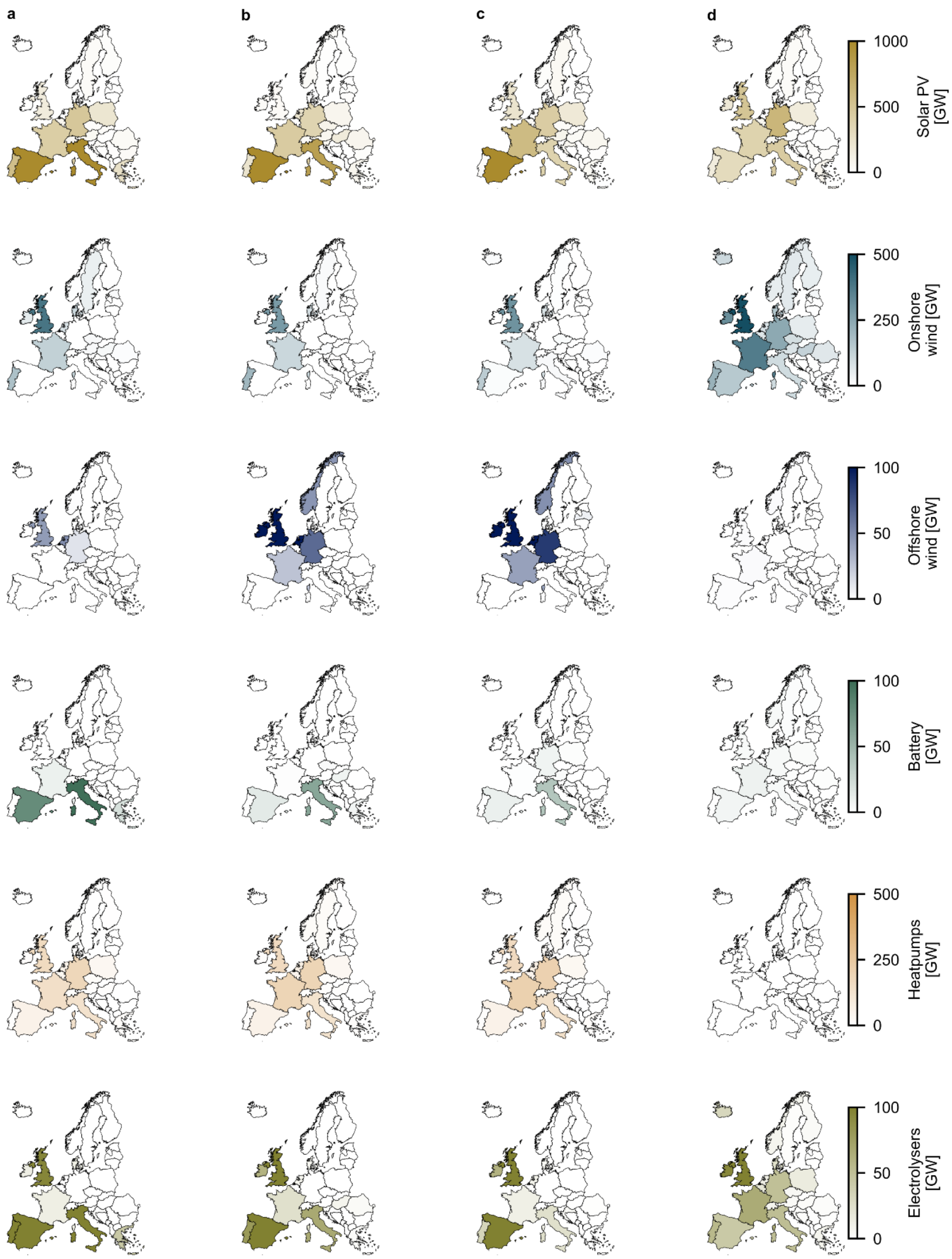

Supplementary Figure 8. Key infrastructure for the top 1% of candidate designs (4 designs) according to indicator 8, maximum job creation (utility). Columns (a-d) present the best-performing candidate designs ordered by rank. Column (a) shows the candidate design that supports the most job creation (Methods – Utilitarian indicators) and column (d) shows the candidate design supporting the fourth-most jobs. Aiming to maximise job creation is the only situation where solar PV is the most important technology in terms of capacity installations (Figure 4). This aim disproportionately benefits southern sunny countries, including Spain (Figure 2).

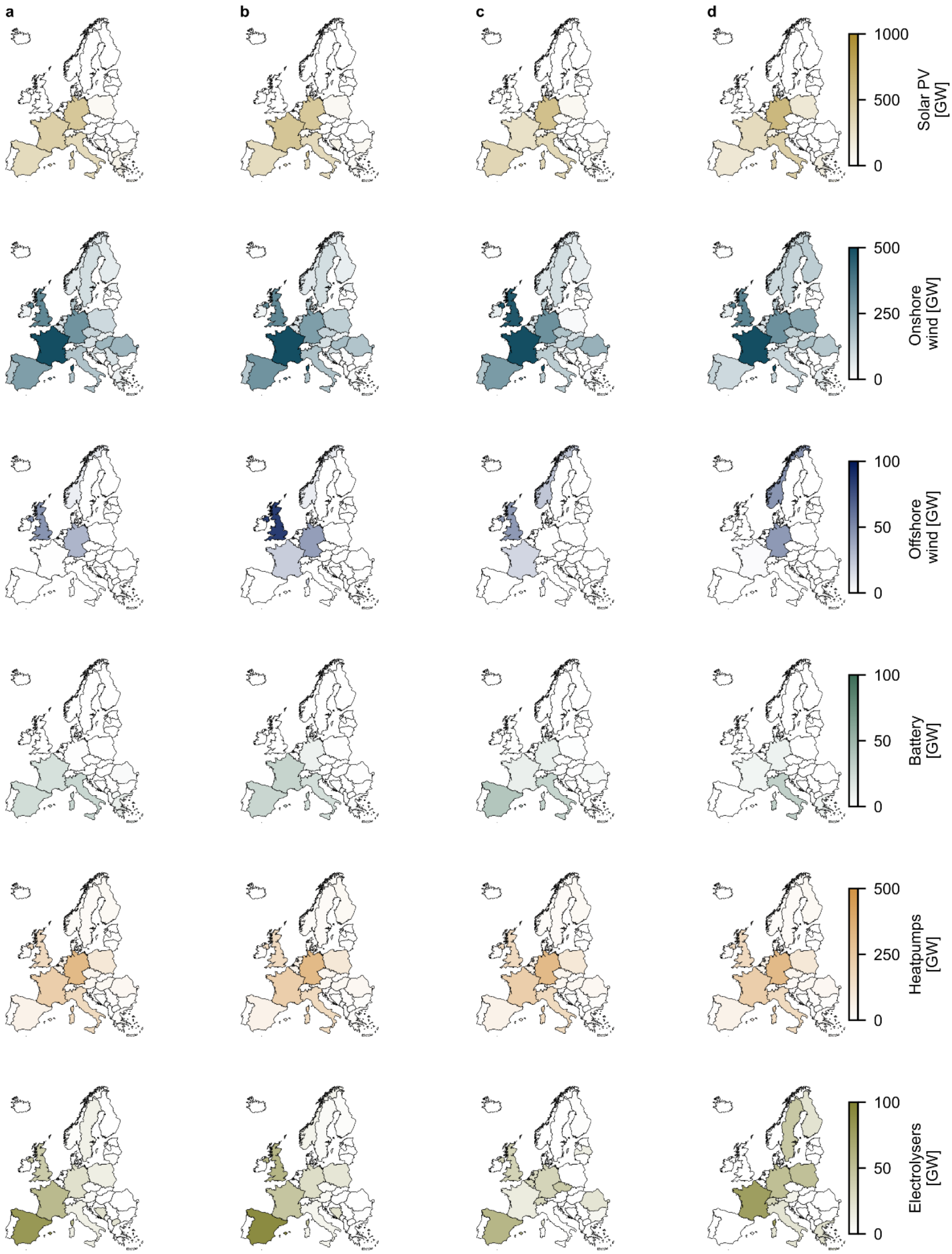

Supplementary Figure 9. Key infrastructure for the top 1% of candidate designs (4 designs) according to indicator 1, equality of investment. Columns (a-d) present the best-performing candidate designs ordered by rank. Column (a) shows the candidate design that supports the most equal investment (Methods – Equality indicators) and column (d) shows the fourth-best-performing candidate design. Note the variable installed capacities battery and electrolyser capacities in Spain.

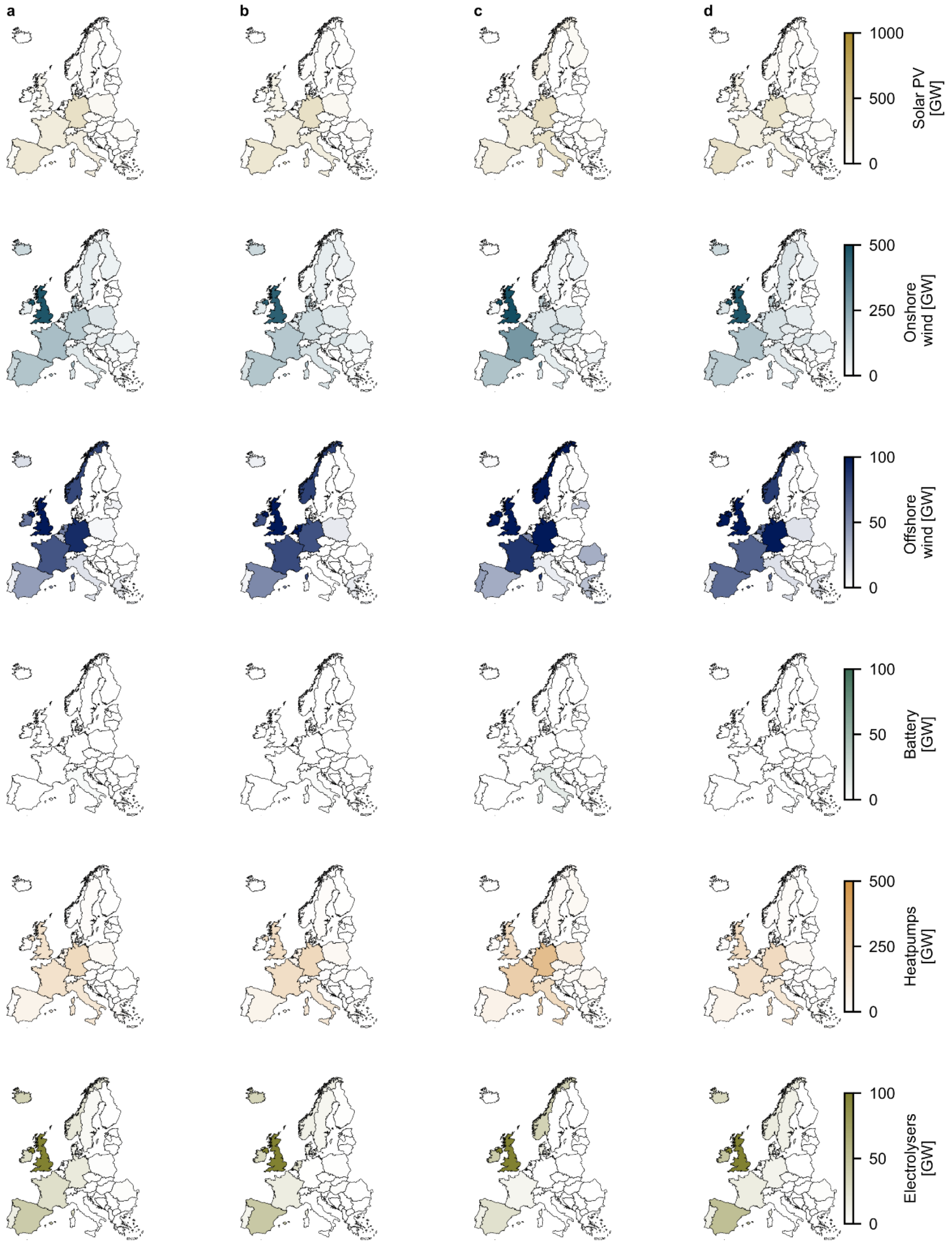

Supplementary Figure 10. Key infrastructure for the top 1% of candidate designs (4 designs) according to indicator 2, equality of job creation. Columns (a-d) present the best-performing candidate designs ordered by rank. Column (a) shows the candidate design that supports the most equal job creation (Methods – Equality indicators) and column (d) shows the fourth-best-performing candidate design. Equal job creation suggests the lowest mean and median battery requirements across all nine indicators.

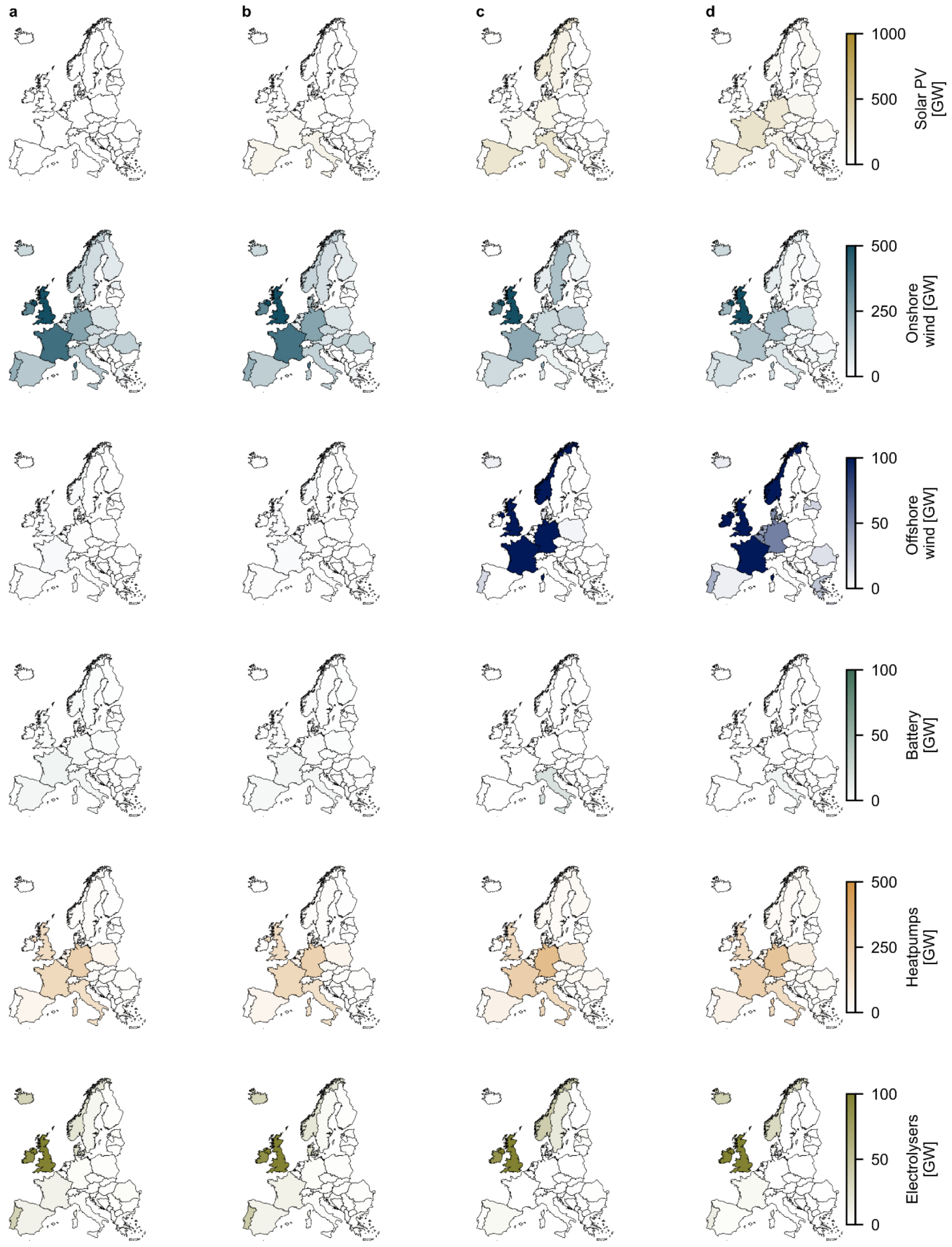

Supplementary Figure 11. Key infrastructure for the top 1% of candidate designs (4 designs) according to indicator 3, equality of land use. Columns (a-d) present the best-performing candidate designs ordered by rank. Column (a) shows the candidate design that supports the most equal distribution of energy-related land use (Methods – Equality indicators) and column (d) shows the fourth-best-performing candidate design. Note the high variability in solar PV and offshore wind requirements, which includes recommended candidate designs featuring lower capacity installations than already installed.

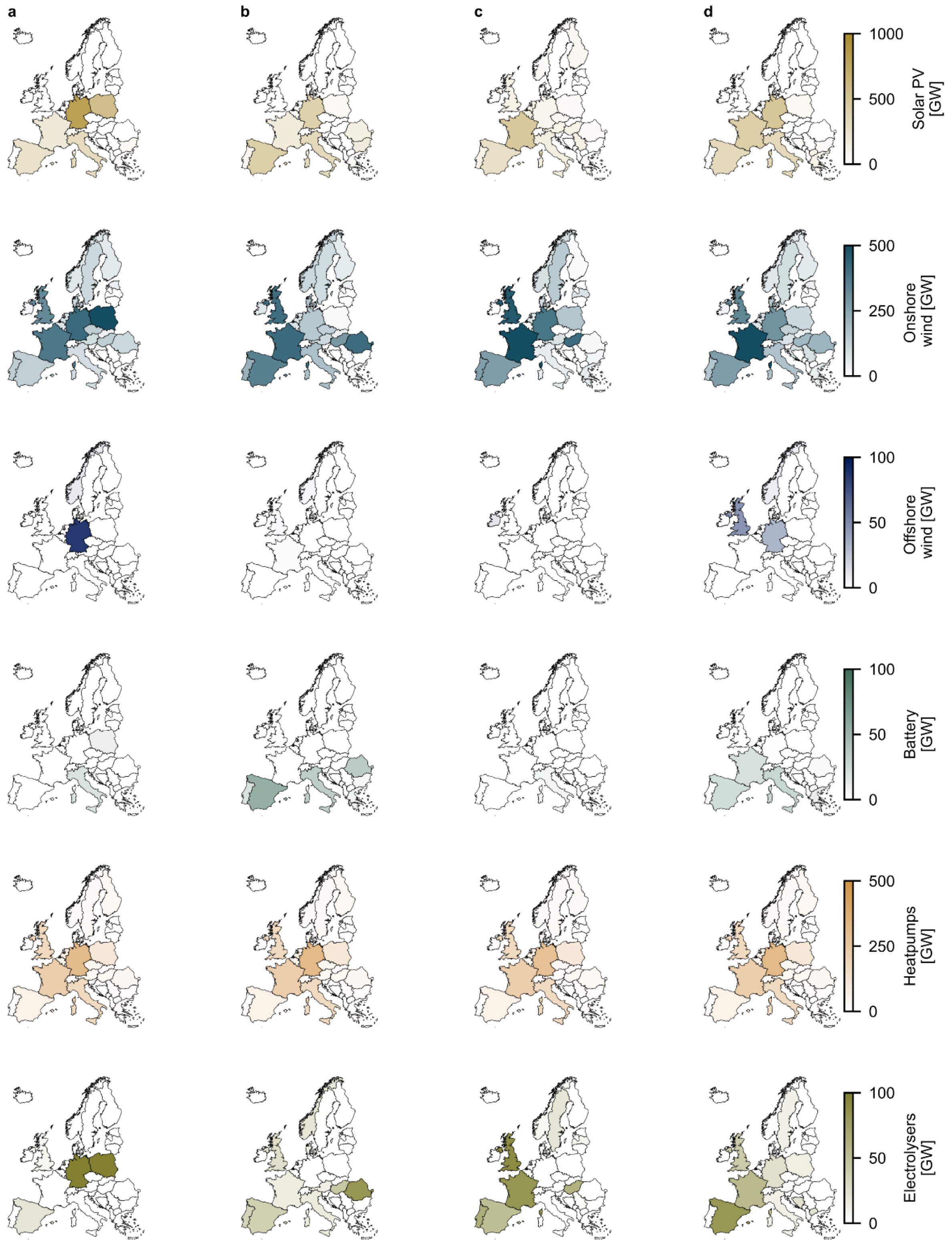

Supplementary Figure 12. Key infrastructure for the top 1% of candidate designs (4 designs) according to indicator 4, equitable investment. Columns (a-d) present the best-performing candidate designs ordered by rank. Column (a) shows the candidate design that supports the most equitable investment distribution according following the principles set out in the Social Cohesion Fund (Methods – Equity indicators) and column (d) shows the fourth-best-performing candidate design. Except for heat pumps, the most important country in terms of capacity installation varies across each of the four recommended candidate designs. Nonetheless, the continent-wide capacity installation ranges are among the smallest across all indicators (Figure 4).

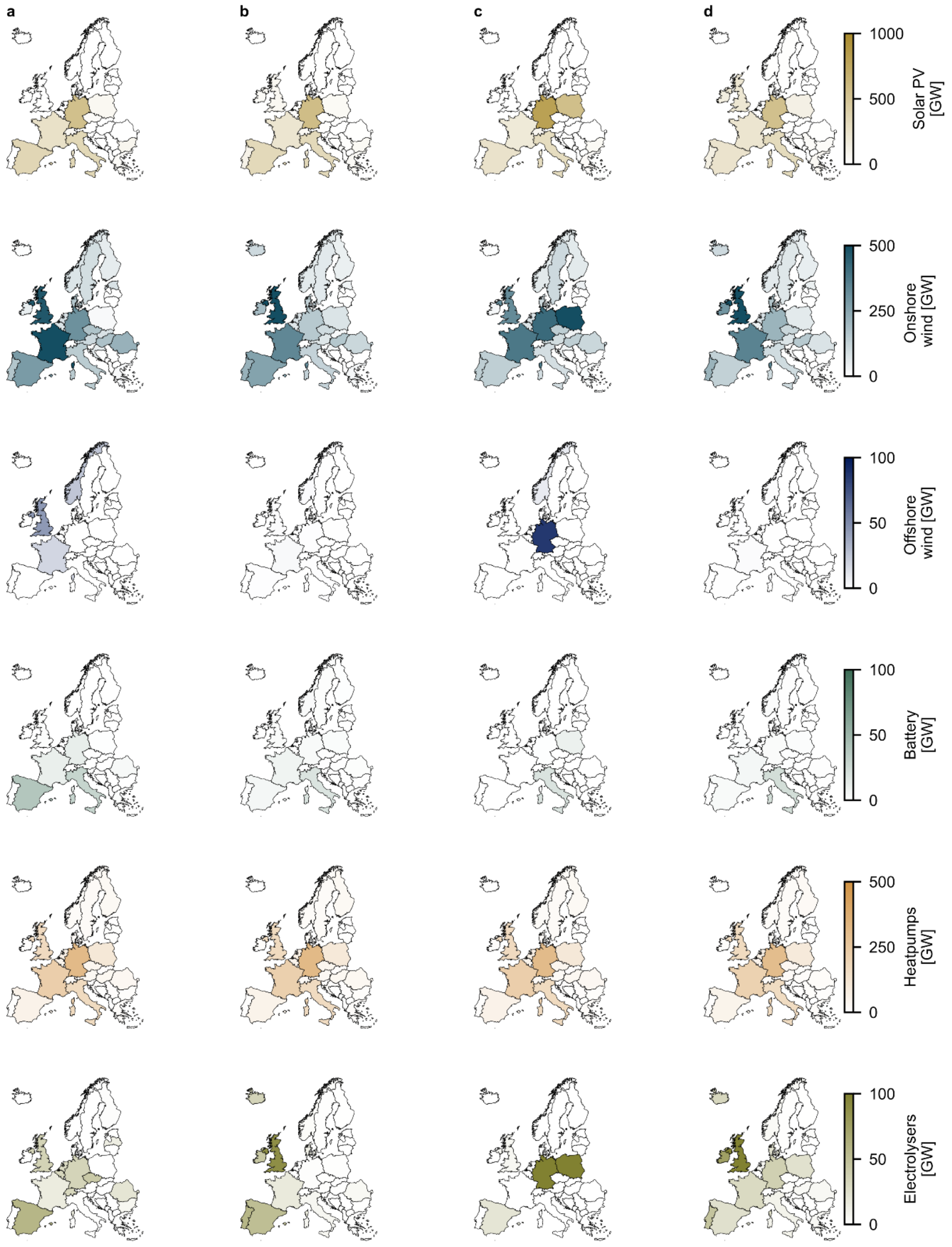

Supplementary Figure 13. Key infrastructure for the top 1% of candidate designs (4 designs) according to indicator 5, equitable job creation. Columns (a-d) present the best-performing candidate designs ordered by rank. Column (a) shows the candidate design that supports the most equitable job creation according to the current distribution of fossil fuel workers (Methods – Equity indicators) and column (d) shows the fourth-best-performing candidate design. The potential country-to-country distribution of offshore wind varies considerably, but the total installation requirements relative to current installed capacity varies by only a factor of 2. Electrolyser capacity is more uncertain in terms of total requirements, with the candidate designs suggesting increases of 700 to over 1,000 times current installed capacity.

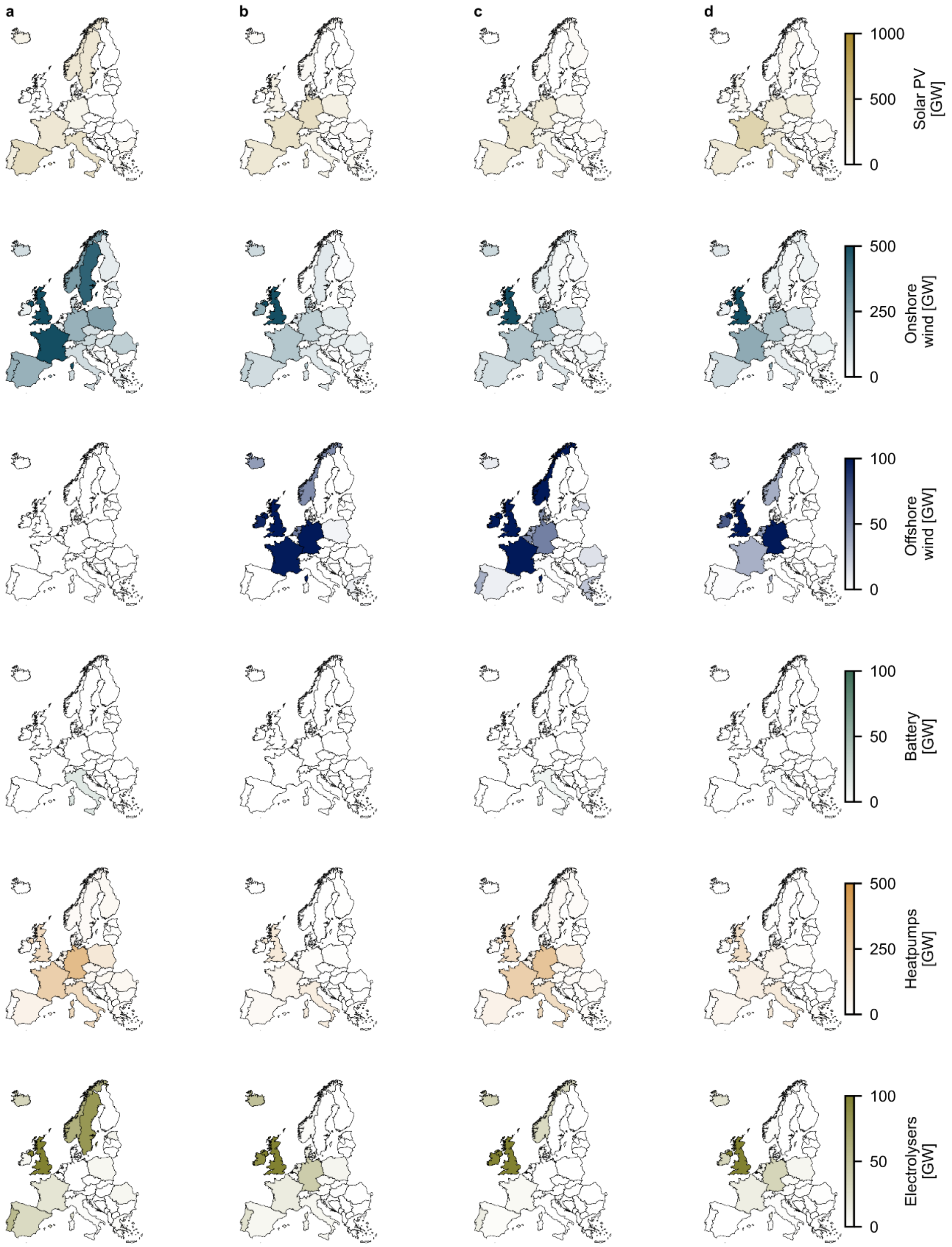

Supplementary Figure 14. Key infrastructure for the top 1% of candidate designs (4 designs) according to indicator 6, equitable land use. Columns (a-d) present the best-performing candidate designs ordered by rank. Column (a) shows the candidate design that supports the most equitable energy-related land use when considering additional protection measures needed to achieve 30% land protection (Methods – Equality indicators) and column (d) shows the fourth-best-performing candidate design. The designs concentrate installed capacity according to available land, with the gradient between larger and smaller countries most visible for the case of offshore wind. The distribution of technologies requiring little-to-no land use are less meaningful for determining the best candidate designs according to this interpretation for distributive justice.

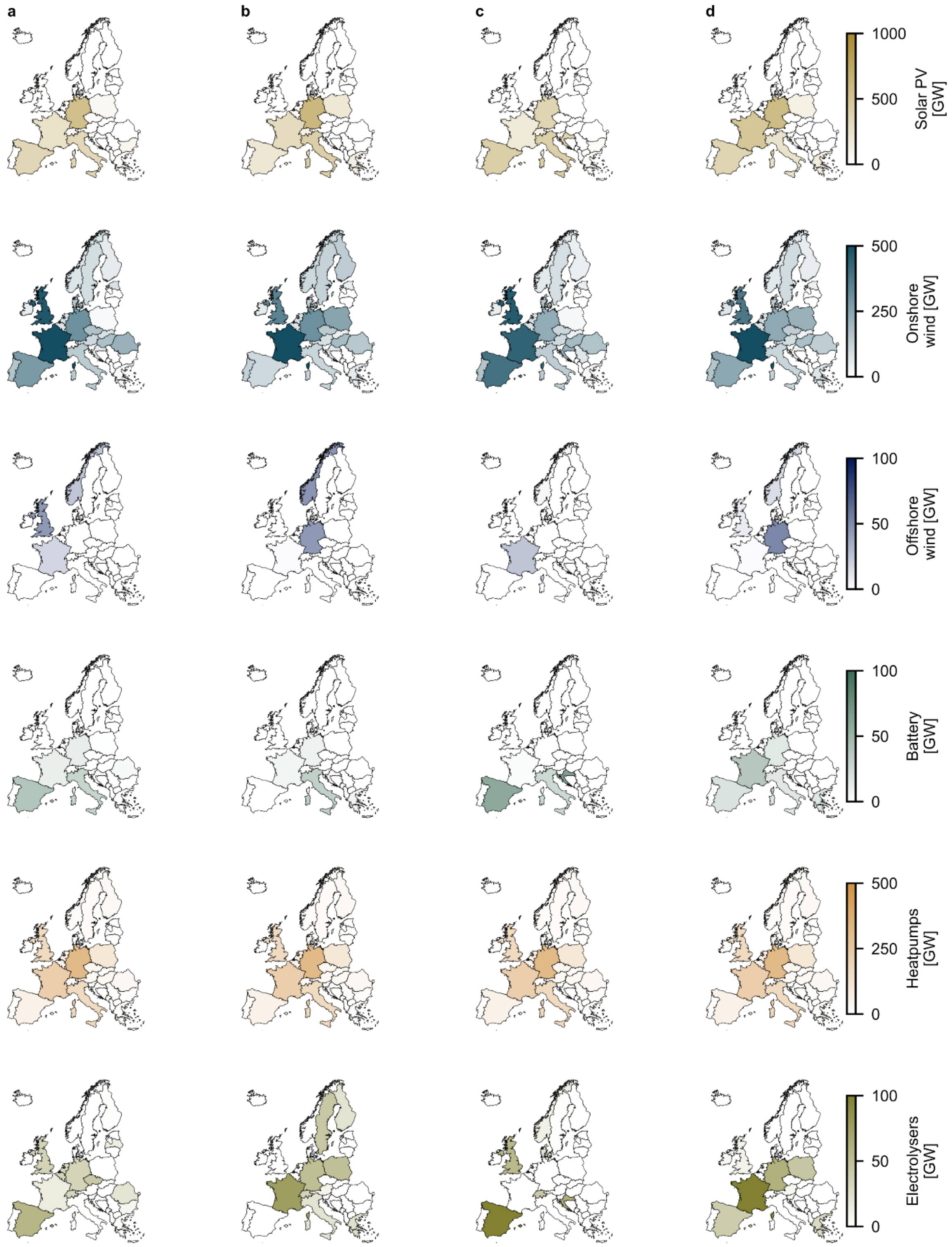

Supplementary Figure 15. Key infrastructure for the top 1% of candidate designs (4 designs) according to indicator 7, minimum investment (utility). Columns (a-d) present the best-performing candidate designs ordered by rank. Column (a) shows the candidate design that supports the lowest investment costs (Methods – Utilitarian indicators) and column (d) shows the fourth-cheapest candidate design. The total variation in absolute capacities is smaller for this indicator than for other indicators (Figure 5), however, national-level installations still vary considerably for some technologies. For example, French electrolyser capacities range from 240 megawatts to 132 gigawatts.

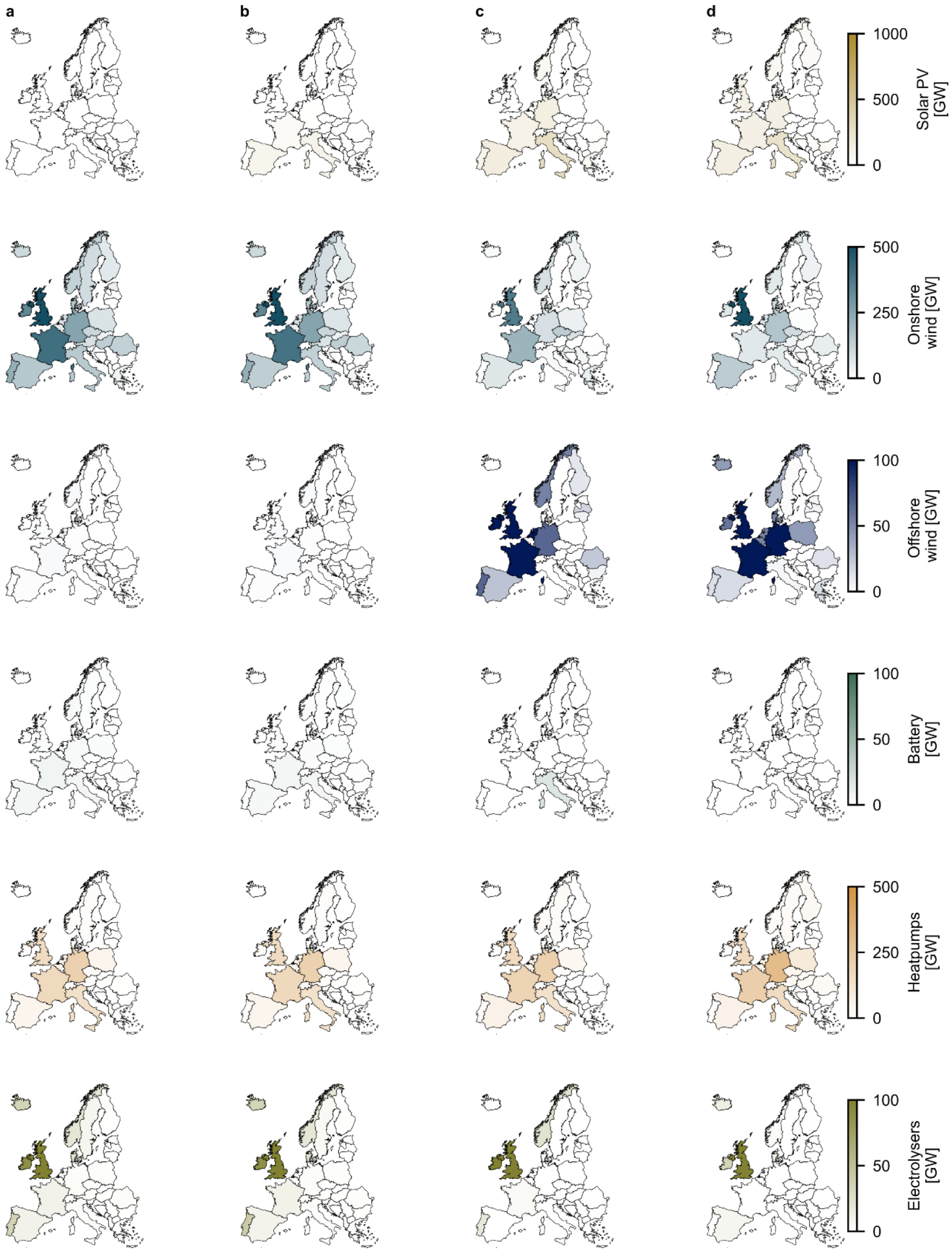

Supplementary Figure 16. Key infrastructure for the top 1% of candidate designs (4 designs) according to indicator 9, minimum land use (utility). Columns (a-d) present the best-performing candidate designs ordered by rank. Column (a) shows the candidate design that supports the lowest energy-related land use (Methods – Utilitarian indicators) and column (d) showing the fourth-least land-intensive candidate design. The total mean and median capacity recommendations for solar PV are smaller for this indicator than for any other indicator. This result stems from the land-intensive nature of open-field PV. Mitigating PV-related land use requirements could be achieved by increasing the density of PV production in open-fields or installing PV to only on existing structures, like roofs and façades (Supplementary Fig. 6).

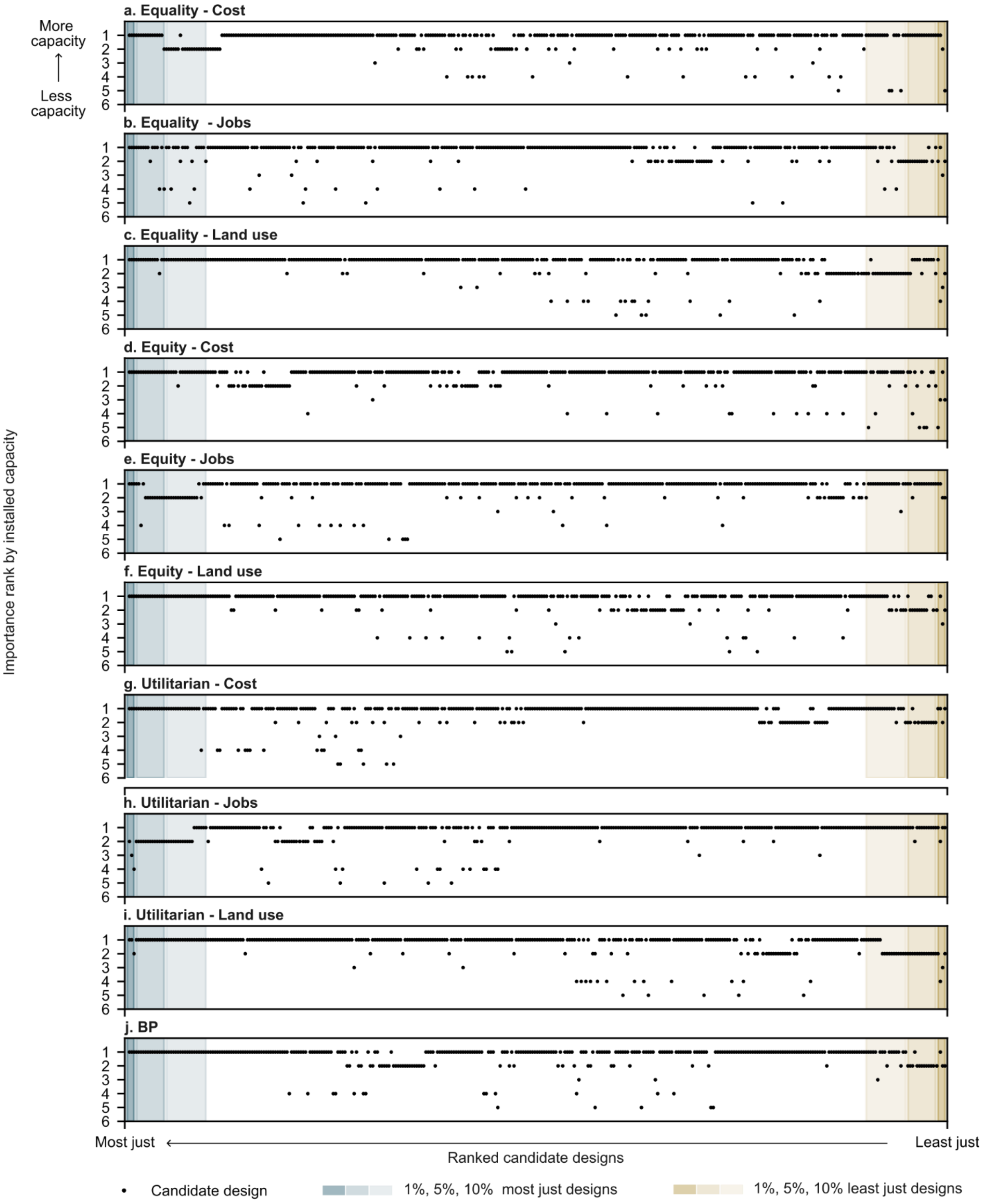

Supplementary Figure 17. Ranked importance of onshore wind capacity versus other key technologies across the justice-ranked candidate designs. Results shown for the nine indicators of distributive justice (a-i) and the BP approach (j). Across all system designs, onshore wind is frequently the most important technology in terms of absolute capacity installation (Rank 1). The technology's relative importance is consistent with the 1% of the most just designs (Figure 5). However, the least just designs can also entail high relative installed onshore wind capacities (e.g., g, h). As such, achieving distributionally just energy systems requires comparatively high shares of onshore wind, but high shares of onshore wind do not necessarily signal just energy systems. Also see Supplementary Fig. 18.

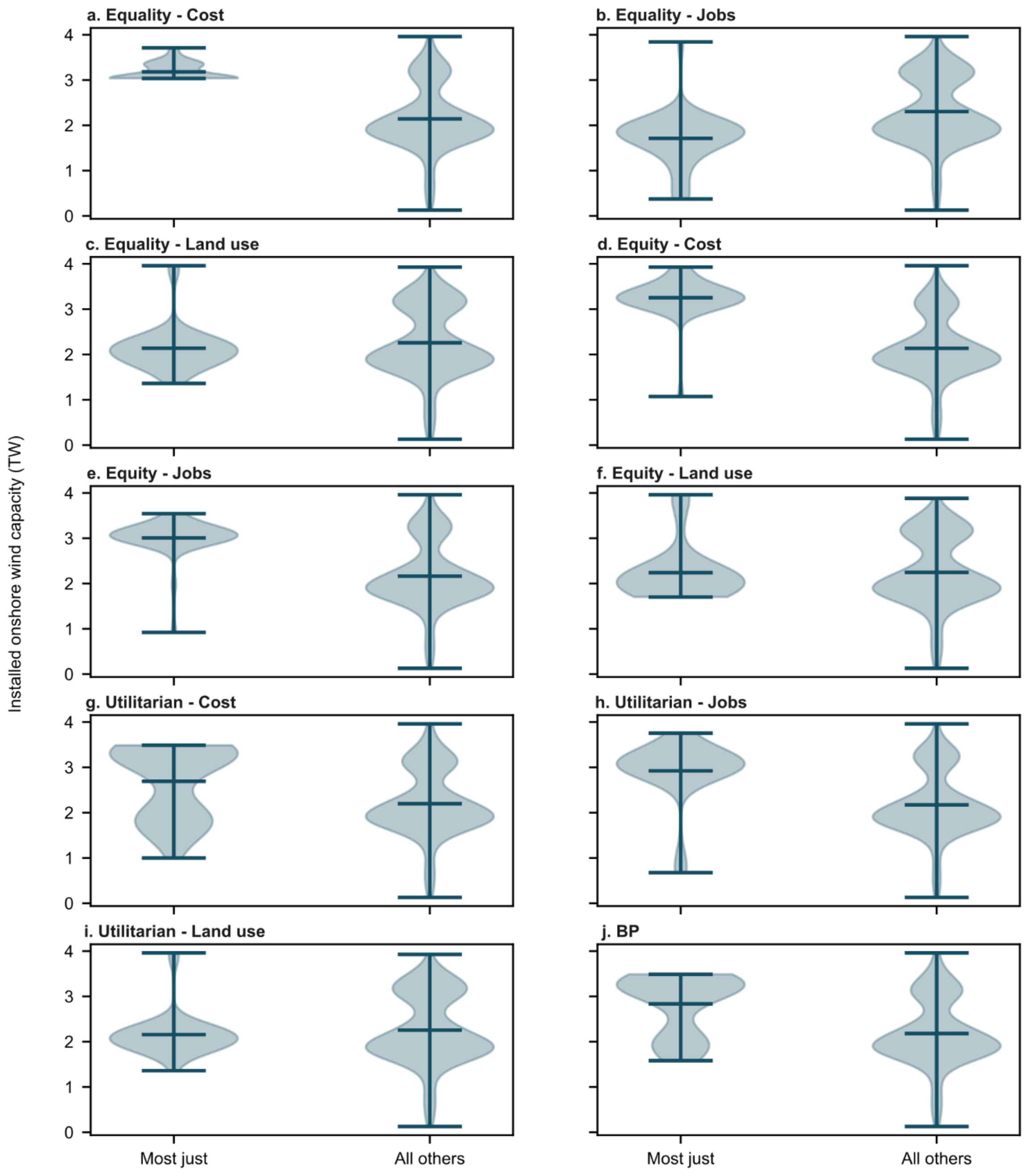

Supplementary Figure 18. Onshore wind capacity distribution of top 10% most just candidate designs ($n_{Most\ just}$=35, n_{Other}=318) according to the nine indicators for distributive justice (a-i) and the BP approach (j). There are statistically significant differences according to the Mann-Whitney-Wilcoxon test in recommended installed onshore capacity for some indicators (a, b, d, e, g, and h). See Supplementary Data for statistical results.

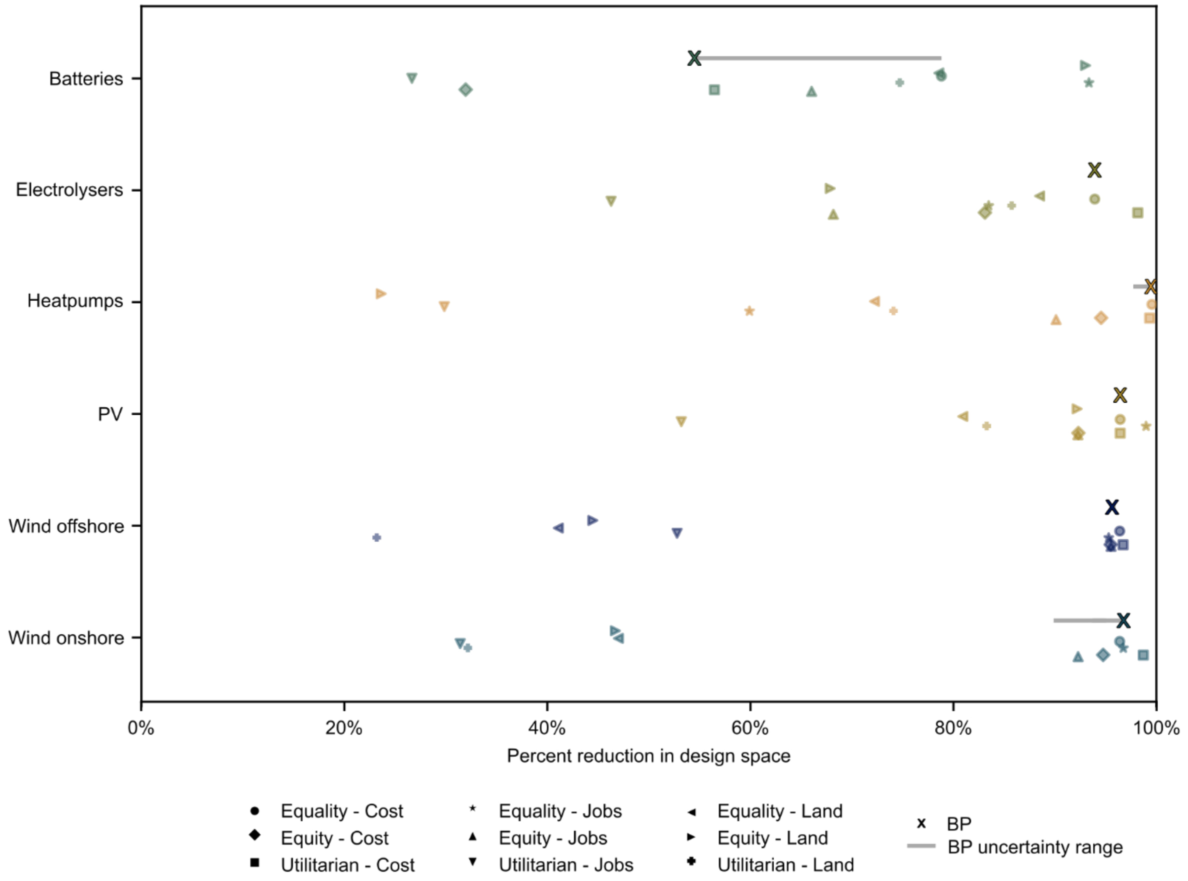

Supplementary Figure 19. Sensitivity analysis on design space reduction by technology. Each symbol corresponds to an indicator for distributive justice. The main results for a balanced priorities (BP) approach are shown alongside the range of possible outcomes considering variability in public preferences. The sensitivity analysis involves randomly sampling preferences from a uniform distribution within the ranges given by sampled European population for different energy system impacts and theories of distributive justice (see Supplementary Table 3). Results are stable except batteries, the uncertainty of which mirrors the real-world uncertainty surrounding how much energy storage is desirable for Europe[7].

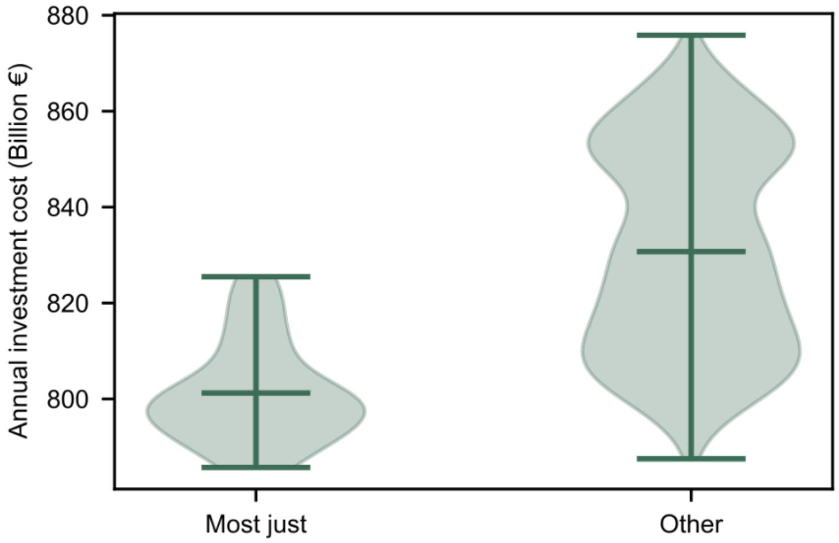

Supplementary Figure 20. Cost distribution of 10% best-scoring BP candidate designs versus all other candidate designs ($n_{Most\ just}$=35, n_{Other}=318). The cost distribution associated with the most just candidate designs is significantly lower than the cost of all other candidate designs according to the Mann-Whitney-Wilcoxon test. The distribution is generated using the Scott kernel density estimator, as implemented in matplotlib violinplot. Center line, mean; whiskers, minimum and maximum.

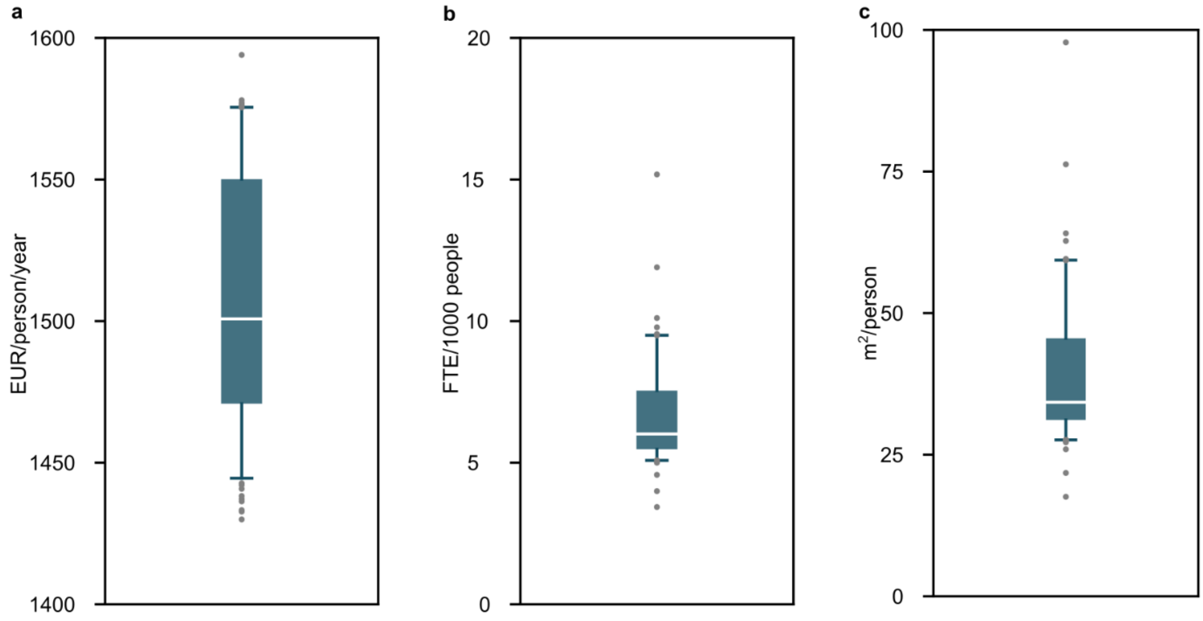

Supplementary Figure 21. Per capita impacts of all 353 unique candidate designs (n=353). Results for (a) per capita investment requirements, (b) job creation potential, and (c) land use requirements. Centre line, median; box limits, upper and lower quartiles; whiskers, 95% of all data; points, outliers.

Supplementary References

1. European Commission, Directorate-Generate for Energy (2019). Special Eurobarometer 492: Europeans' attitudes on EU energy policy (European Commission Directorate-General for Communication).

2. European Commission, Directorate-General for Employment, Social Affairs and Inclusion (2022). Special Eurobarometer 527: Fairness perceptions of the green transition (European Commission Directorate-General for Communication).

3. European Commission. Directorate General for Environment. (2024). Special Eurobarometer 550: Attitudes of Europeans towards the environment (Publications Office).

4. Virtanen, P., Gommers, R., Oliphant, T.E., Haberland, M., Reddy, T., Cournapeau, D., Burovski, E., Peterson, P., Weckesser, W., Bright, J., et al. (2020). SciPy 1.0: fundamental algorithms for scientific computing in Python. Nat. Methods *17*, 261–272. https://doi.org/10.1038/s41592-019-0686-2.

5. Lonergan, K.E., and Sansavini, G. (2025). Considering distributive justice as a planning principle helps navigate a diversity of future energy infrastructure designs. (Code Ocean). https://doi.org/10.24433/CO.2884991.v1 https://doi.org/10.24433/CO.2884991.v1.

6. ecoinvent ecoinvent database. https://ecoinvent.org/database/ https://ecoinvent.org/database/.

7. ENTSO-e (2023). System needs study: opportunities for a more efficient European power system in 2030 and 2040 (European Network of Transmission System Operators for Electricity).
