# Supplementary material for: Considering distributive justice as a planning principle helps navigate a diversity of future energy infrastructure designs
Source: Nat Commun. 2025 Nov 25;16:10509. doi: 10.1038/s41467-025-65526-0 (PMC12647790; doi:10.1038/s41467-025-65526-0)
Supplement: Supplementary file 2 — Description of Addtional Supplementary File [file 41467_2025_65526_MOESM2_ESM.pdf]

Description of Additional Supplementary Data File

Supplementary Data 1. Data underlying the figures in the main text.
