# Supplementary material for: Considering distributive justice as a planning principle helps navigate a diversity of future energy infrastructure designs
Source: Nat Commun. 2025 Nov 25;16:10509. doi: 10.1038/s41467-025-65526-0 (PMC12647790; doi:10.1038/s41467-025-65526-0)
Supplement: Supplementary file 4 — Reporting Summary [file 41467_2025_65526_MOESM4_ESM.pdf]

nature portfolio

Corresponding author(s): Giovanni Sansavini (sansavig@ethz.ch)

Last updated by author(s): Sep 22, 2025

Reporting Summary

Nature Portfolio wishes to improve the reproducibility of the work that we publish. This form provides structure for consistency and transparency in reporting. For further information on Nature Portfolio policies, see our Editorial Policies and the Editorial Policy Checklist.

Statistics

For all statistical analyses, confirm that the following items are present in the figure legend, table legend, main text, or Methods section.

n/a	Confirmed	
☐	☒	The exact sample size (*n*) for each experimental group/condition, given as a discrete number and unit of measurement
☐	☒	A statement on whether measurements were taken from distinct samples or whether the same sample was measured repeatedly
☐	☒	The statistical test(s) used AND whether they are one- or two-sided<br>*Only common tests should be described solely by name; describe more complex techniques in the Methods section.*
☒	☐	A description of all covariates tested
☐	☒	A description of any assumptions or corrections, such as tests of normality and adjustment for multiple comparisons
☐	☒	A full description of the statistical parameters including central tendency (e.g. means) or other basic estimates (e.g. regression coefficient) AND variation (e.g. standard deviation) or associated estimates of uncertainty (e.g. confidence intervals)
☐	☒	For null hypothesis testing, the test statistic (e.g. *F*, *t*, *r*) with confidence intervals, effect sizes, degrees of freedom and *P* value noted<br>*Give P values as exact values whenever suitable.*
☒	☐	For Bayesian analysis, information on the choice of priors and Markov chain Monte Carlo settings
☒	☐	For hierarchical and complex designs, identification of the appropriate level for tests and full reporting of outcomes
☐	☒	Estimates of effect sizes (e.g. Cohen's *d*, Pearson's *r*), indicating how they were calculated

Our web collection on statistics for biologists contains articles on many of the points above.

Software and code

Policy information about availability of computer code

Data collection	This analysis relies entirely on open data. All data needed to perform the analysis is available on Code Ocean (https://doi.org/10.24433/CO.2884991.v1).
Data analysis	o The Python (3.10) and RMarkdown (4.0.2) code used for this analysis is available on Code Ocean (https://doi.org/10.24433/CO.2884991.v1). All maps have been generated using data files from the World Food Programme and available via opendatasoft (https://public.opendatasoft.com/explore/dataset/world-administrative-boundaries/information/?refine.continent=Europe) and modified using QGIS (http://qgis.org). The colour scheme was developed by Fabio Crameri (https://doi.org/10.5281/zenodo.1243862). Analysis was performed using PyCharm Professional (2025.1) using an academic license (https://www.jetbrains.com/pycharm/).

For manuscripts utilizing custom algorithms or software that are central to the research but not yet described in published literature, software must be made available to editors and reviewers. We strongly encourage code deposition in a community repository (e.g. GitHub). See the Nature Portfolio guidelines for submitting code & software for further information.

Data

Policy information about availability of data

All manuscripts must include a data availability statement. This statement should provide the following information, where applicable:

- Accession codes, unique identifiers, or web links for publicly available datasets
- A description of any restrictions on data availability
- For clinical datasets or third party data, please ensure that the statement adheres to our policy

• This analysis relies entirely on openly available data. The data used in this study are available in the Code Ocean database (https://doi.org/10.24433/CO.2884991.v1). The data generated in this study are provided in Zenodo (www.doi.org/10.5281/zenodo.17177324). and available via opendatasoft (https://public.opendatasoft.com/explore/dataset/world-administrative-boundaries/information/?refine.continent=Europe) and modified using QGIS (http://qgis.org). The colour scheme was developed by Fabio Crameri (https://doi.org/10.5281/zenodo.1243862). Analysis was performed using PyCharm Professional (2025.1) using an academic license (https://www.jetbrains.com/pycharm/).

Research involving human participants, their data, or biological material

Policy information about studies with human participants or human data. See also policy information about sex, gender (identity/presentation), and sexual orientation and race, ethnicity and racism.

Reporting on sex and gender	N/A
Reporting on race, ethnicity, or other socially relevant groupings	N/A
Population characteristics	N/A
Recruitment	N/A
Ethics oversight	N/A

Note that full information on the approval of the study protocol must also be provided in the manuscript.

Field-specific reporting

Please select the one below that is the best fit for your research. If you are not sure, read the appropriate sections before making your selection.

☐ Life sciences ☒ Behavioural & social sciences ☐ Ecological, evolutionary & environmental sciences

For a reference copy of the document with all sections, see nature.com/documents/nr-reporting-summary-flat.pdf

Behavioural & social sciences study design

All studies must disclose on these points even when the disclosure is negative.

Study description	We rely on results from three recent Eurobarometer surveys relevant to understanding the opinions of Europeans towards the development of fair energy systems. The surveys are: (1) Special Eurobarometer 492: European attitudes on EU Energy Policy (SP42), (2) Special Eurobarometer 527: Fairness perceptions of the green transition, and (3) Special Eurobarometer 550: Attitudes of Europeans towards the Environment. The surveys contain a mix of qualitative and quantitative data.
Research sample	All respondents must have been be 15 or older to complete the survey. The three surveys each had over 25 000 respondents: SP492: 27 438 respondents across the EU28, SP527: 26 395 across the EU27, and SP550: 26 346 across the EU27.
Sampling strategy	Participants were identified using the common, multi-stage probability-based sampling strategy applied to all Eurobarometer surveys. First-stage sample points were drawn from regional administrative units and degree of urbanization. Second-stage sample points were drawn from the population size, before randomly sampling a coordinate and then identifying the nearest closest address. Further addresses were selected following “random route” procedures. The procedure of identifying participants was modified slightly for the Netherlands, Finland, and Sweden to randomly select addresses from within the population, telephone, and address registers. The data collection teams differ from the analysis teams; their awareness of the study hypotheses is unknown.
Data collection	The surveys are offered in the national language(s) at home, via online forms or face-to-face or telephone interviews. The surveys are conducted face-to-face, via telephone, and online, partially as a response to COVID-19 health measures. The majority of questions are formulated as multiple choice questions, allowing respondents “totally agree”, “tend to agree”, “agree”, “disagree”, “tend to disagree”, “totally disagree”, or give no opinion to a policy statement. If no contact was made at a given household, the interviewer would revisit the household for an additional three attempts.

Timing	The fieldwork for the three surveys are as follows: SP492: 09.05.2019-25.05.2019, SP527: 30.05.2022-28.06.2022, and SP550: 06.03.2024-08.04.2024.
Data exclusions	Only relevant data from the Eurobarometer surveys was included in the present analysis, i.e., results from irrelevant questions were not included. No data from relevant survey questions was excluded from the present study.
Non-participation	Results from the United Kingdom and Northern Ireland are unavailable for SB527 and SB550 due to the UK&NI leaving the European Union. Some countries considered in the present study (Albania, Bosnia and Herzegovina, Iceland, North Macedonia, Montenegro, Norway, Serbia, and Switzerland), were not polled within the survey process because they are not part of the European Union.
Randomization	The surveys randomly polls at least 1,000 people per country. A sample size of 500 persons is used in countries or territories with a population of below one million inhabitants. Participants are randomly selected and the total sample is weighted to ensure demographic and geographical representativeness.

Reporting for specific materials, systems and methods

We require information from authors about some types of materials, experimental systems and methods used in many studies. Here, indicate whether each material, system or method listed is relevant to your study. If you are not sure if a list item applies to your research, read the appropriate section before selecting a response.

Materials & experimental systems

n/a	Involved in the study
☒	☐ Antibodies
☒	☐ Eukaryotic cell lines
☒	☐ Palaeontology and archaeology
☒	☐ Animals and other organisms
☒	☐ Clinical data
☒	☐ Dual use research of concern
☒	☐ Plants

Methods

n/a	Involved in the study
☒	☐ ChIP-seq
☒	☐ Flow cytometry
☒	☐ MRI-based neuroimaging

Plants

Seed stocks	N/A
Novel plant genotypes	N/A
Authentication	N/A
